# Unbiased Metabolomics Links Fatty Acid Pathways to Psychiatric Symptoms in People Living with HIV

**DOI:** 10.3390/jcm10235466

**Published:** 2021-11-23

**Authors:** Elise Meeder, Vasiliki Matzaraki, Nadira Vadaq, Lisa van de Wijer, André van der Ven, Arnt Schellekens

**Affiliations:** 1 Department of Psychiatry, Radboud University Medical Centre, 6525 GA Nijmegen, The Netherlands; Arnt.Schellekens@radboudumc.nl; 2 Nijmegen Institute for Scientist-Practitioners in Addiction (NISPA), Radboud University, 6500 HE Nijmegen, The Netherlands; 3 Donders Institute for Brain, Cognition and Behavior, Radboud University, 6525 AJ Nijmegen, The Netherlands; 4 Department of General Internal Medicine, Radboud University Medical Centre, 6525 GA Nijmegen, The Netherlands; Vasiliki.Matzaraki@radboudumc.nl (V.M.); nadiravadaq@gmail.com (N.V.); L.vandeWijer@radboudumc.nl (L.v.d.W.); Andre.vanderVen@radboudumc.nl (A.v.d.V.); 5 Center for Tropical and Infectious Diseases (CENTRID), Faculty of Medicine, Diponegoro University, Dr. Kariadi Hospital, Semarang 1269, Indonesia

**Keywords:** HIV, metabolome, addiction, depression, impulsivity

## Abstract

Psychiatric symptoms are prevalent in people living with HIV (PLWH), especially depression, anxiety, impulsivity, and substance use. Various biological mechanisms might play a role in the occurrence of psychiatric symptoms in this population. A hypothesis free, data-driven metabolomics approach can further our understanding of these mechanisms. In this study, we identified metabolic pathways associated with impulsivity, depression and substance use in 157 PLWH. First, Spearman’s rank correlations between metabolite feature intensities and psychiatric symptom levels were calculated, while controlling for age, gender and body mass index. Subsequently, a mummichog pathway analysis was performed. Finally, we analyzed which individual metabolites drove the observed effects. In our cohort of PLWH, fatty acid-related pathways were associated with both depressive as well as impulsive symptomatology. Substance use showed most extensive metabolic associations, and was positively associated with short chain fatty acids (SCFA’s), and negatively associated with glutamate levels. These findings suggest that PUFA metabolism might be associated with both internalising and externalising symptomatology in PLWH. Furthermore, glutamate and SCFA’s—microbiome derivatives with known neuroactive properties—might be involved in substance use in these patients. Future studies should explore potential causal mechanisms involved and whether these findings are HIV-specific.

## 1. Introduction

Human immunodeficiency virus (HIV) infection is a continuing global public health threat, with an annual incidence of about 1.7 million individuals [1]. The introduction of combination antiretroviral therapy (cART) has converted life expectancy of people living with HIV (PLWH) to near normal levels. Consequently, quality of life has become an increasingly important topic [2]. A major factor in perceived quality of life in PLWH is psychiatric symptomatology, which is more prevalent in PLWH than in the general population [3,4]. Stress and impulsivity-related disorders are common in PLWH, as is reflected by elevated prevalence rates of depression (around 10%, versus 5% in the general population), and alcohol use disorder (up to 40% in HIV-related primary care, versus 20–30% in the general primary care setting) [5].

Several mechanisms might be involved in the frequent psychiatric symptomatology in PLWH [6,7]. Psychiatric symptoms might be a premorbid risk factor for acquiring HIV, e.g., through increased transmission risk behaviour in people with high impulsive traits [8]. In addition, psychological mechanisms might play an important role, for instance due to increased stress, associated with receiving an HIV diagnosis, or stigma related to HIV [9,10]. However, also biological mechanisms might play a role. For example, HIV has been shown to induce neurotoxicity in dopaminergic neurons, and decrease dopamine sensitivity, which has among others been associated with ADHD and vulnerability for addictive behaviours [11,12].

Though several studies explored potential biological mechanisms involved in psychiatric symptomatology in PLWH, these studies commonly explore a single set of psychiatric symptoms, in relation to a single set of biological markers. Such studies have suggested a role of pro-inflammatory cytokines, hypothalamic-pituitary-adrenal (HPA-) axis activation, and decreased levels of brain derived neurotrophic factor (BDNF) in depressive symptoms in PLWH, and dopamine transporter disfunction in substance use in PLWH [13,14,15,16]. Consequently, multiple pathophysiological mechanisms might be involved in psychiatric symptomatology in PLWH. Furthermore, there is increasing evidence that different psychiatric symptoms might share common pathophysiological mechanisms. For example, glutamate system alterations have been related to depression, anxiety, impulsivity and substance abuse [17,18,19,20]. In order to gain a better understanding of the biological mechanisms involved in psychiatric symptomatology in PLWH, a variety of biological factors and psychiatric symptoms should be explored simultaneously.

Recently, untargeted, data-driven approaches have attracted scientific interest because such approaches enable the investigation of multiple systems simultaneously. An example of such untargeted research is metabolomics, which systematically identifies and quantifies all metabolites present in a biofluid or tissue [21]. This technique allows unbiased assessment of numerous biological pathways on a highly detailed, functional level. This approach has shown limited application to psychiatric symptomatology in PLWH. One study showed an association between monoamine and acylcarnitine metabolites with depression in two small cohorts of 32 and 36 PLWH [22]. However, it should be noted that a substantial number of patients was not receiving cART, while some cART receiving subjects were using antiretroviral drugs with known psychiatric side effects, and significant mitochondrial toxicity, which might have interfered with acylcarnitine alterations [23]. A second study in two cohorts of 55 and 44 PLWH identified lower steroids to be associated with depression [24]. In this study, patients with mild symptoms were excluded, thus ignoring a major part of the depression continuum.

The aim of the current study is to evaluate metabolic pathways involved in psychiatric symptomatology in PLWH. Using a data-driven, metabolome wide approach, we first identified which metabolic pathways were associated with symptoms of depression, impulsivity and substance use. Next, we looked in more detail which metabolites drove these effects.

## 2. Materials and Methods

### 2.1. Design

In a cross-sectional design, associations between metabolite profiles and depressive, impulsive and substance use symptomatology were assessed in a group of PLWHs. The present study is part of the Human Functional Genomics Project (HFGP) (http://www.humanfunctionalgenomics.org, accessed on 18 October 2021) [25].

### 2.2. Participants

PLWH were recruited from the HIV clinic of Radboud University Medical Centre between 14 December 2015 and 6 February 2017, as has been described before [26]. Inclusion criteria were age ≥18 years, Caucasian ethnicity, HIV-RNA levels <200 copies/mL and receiving cART >6 months. Exclusion criteria were any signs of acute or opportunistic infections, active hepatitis B/C or antibiotic use within the month prior to study visit. See Supplementary Figure S1 for the study flow-chart.

### 2.3. Instruments

#### 2.3.1. Clinical Measures

Sociodemographic and clinical data were collected using Castor Electronic Data Capture program (Castor EDC, CIWIT B.V., Amsterdam, The Netherlands) and extracted from clinical files.

Symptoms of depression, anxiety and stress were measured using the Depression Anxiety Stress Scale 42 (DASS-42). The DASS-42 is a self-report questionnaire consisting of three subscales, with each subscale containing 14 questions [27]. Patients rated the degree to which each item applied to them over the past week, using a 4-point Likert-type scale that ranges from 0 (“Did not apply to me at all”) to 3 (“Applied to me very much, or most of the time”). Scores were calculated by summing the item scores. Subscale scores range from 0 to 42 and the total score from 0 to 126 [27]. The DASS was shown to have excellent psychometric properties in both clinical and nonclinical populations; it has been demonstrated to have adequate validity and high internal consistency [27,28,29,30]. Furthermore, the DASS has repeatedly been used in previous PLWH studies [31,32].

Impulsivity was assessed through the Barratt Impulsiveness Scale (BIS-11). The BIS-11 is a self-report questionnaire, consisting of 30 items divided over three subscales: attentional, motor and non-planning impulsiveness [33]. Attentional impulsiveness (8 items) reflects the inability to focus and the tendency to make rapid decisions. Motor impulsiveness (11 items) reflects the tendency to act quickly. Non-planning impulsivity (11 items) reflects the inability to plan for the future. Patients rated the frequency in which each item applies to them, using a 4-point Likert scale ranging from 1 (“rarely/never”) to 4 (“almost always”). Subscale scores were calculated by summing the item scores. Higher scores indicate higher impulsivity levels. The BIS-11 has been demonstrated to have a good internal consistency and retest reliability [34]. It is considered the golden standard self-report instrument in the field of impulsivity research, and has been extensively used to study impulsivity, including in PLWH [8,35,36,37].

Substance use was assessed using the Measurements in the Addictions for Triage and Evaluation (MATE)-Q [38]. MATE-Q is a self-report questionnaire, of which parts 1a, 1b, and 2 were used. Part 1a consists of 10 items, exploring use patterns of a specific drug of abuse (e.g., alcohol, nicotine, cannabis, etc). Participants rated if they ever used it, as well as the duration of substance use. In part 1b participants rated the frequency of substance use in the past 30 days, and for alcohol and nicotine also the amount used per day. Part 2 of the MATE-Q is based on to the Obsessive-Compulsive Drinking and drug use Scale (OCDS-5) [39]. This is an optimised, short version of the OCDS, which aims to measure craving [40]. The OCDS-5 has superior validity as compared to the OCDS, and showed good reliability [39,40]. It consists of five items regarding drug use obsessions (intrusive thoughts) and behavioural intensions to use drugs. The total score ranges from 0 to 20 [39]. The MATE 2.1 is available in six languages and is used in multiple European countries [41,42,43]. It has been implemented in Dutch addiction care as part of routine outcome monitoring, and has been shown to have adequate psychometric properties [38]. The self-reported version MATE-Q has been demonstrated to have an acceptable concurrent validity with the MATE 2.1 [44].

#### 2.3.2. Mass Spectrometry and Spectral Data Processing

Untargeted metabolomics was performed by flow injection electrospray– time-of-flight (TOF) mass spectrometry in collaboration with General Metabolics, LLC (Boston, MA, USA)), according to a previously described methodology [45].

Participants were not restricted from eating, drinking, or smoking prior to blood sample collection. Venous blood was collected between 8 and 11 a.m. in sterile 10 mL EDTA BD Vacutainer^®^ tubes (Becton-Dickinson, Franklin Lakes, NJ, USA) and frozen and stored within 1–4 h.

Data were processed using quantile normalization and Pareto scaling. Outliers were detected using principal component analysis (PCA). Data points exceeding three standard deviations (SDs) from the first and second principal component were excluded for subsequent analysis (*n* = 2). In total, 1659 metabolite features were identified.

### 2.4. Statistical Analyses

The population was described using descriptive statistics: for normally distributed, continuous variables means and standard deviations were calculated, for abnormally distributed, continuous variables medians and interquartile ranges were calculated, and for categorical variables, numbers and percentages were calculated.

To assess associations between metabolic profiles and psychiatric symptoms, Spearman’s rank correlation coefficients between the intensities of metabolite features and DASS-42, MATE-Q and BIS-11 scores were calculated using partial correlation as implemented in the “pcor” function of the “ppcor” package of the R programming language. Correlations were controlled for age, gender and body-mass-index (BMI).

To assess if psychiatric symptoms were associated with specific metabolic pathways, the mummichog algorithm was applied using the MS Peaks to Pathways module in the web-based platform MetaboAnalyst 5.0 [46]. Mummichog predicts metabolomic network activity directly from *m*/*z* features, bypassing the bottleneck of metabolite identification [47]. The top 10% most significantly associated *m*/*z* features were used as input to mummichog, and the full list of *m*/*z* features was used as a reference. The mass accuracy was set at 0.05 ppm and the analytical mode was negative. The MFN model was selected as pathway library, which is a human genome-scale metabolic model originating from multiple sources (a.o. KEGG, BiGG and Edinburgh model) [47]. Only metabolic pathways containing at least 3 significant metabolites were included. The significance level for the pathway results was set at *p* < 0.05. R version 3.5.3 (R Core team, Vienna, Austria) was used for all analyses and the pathway analysis was performed using Mummichog version 1.0.10 [47] on the web-based platform MetaboAnalyst 5.0 (www.metaboanalyst.ca).

### 2.5. Data Availability

All data analyzed in this study are available from the corresponding author on reasonable request.

## 3. Results

Demographic and clinical characteristics of the participants are provided in Table 1. The mean age was 51.5 years (±10.7), and a minority was female (8.9%). The most frequent way of HIV transmission was through sexual contact between men who have sex with men (MSM). All were infected with HIV-1 and received cART.

**Figure 1:**
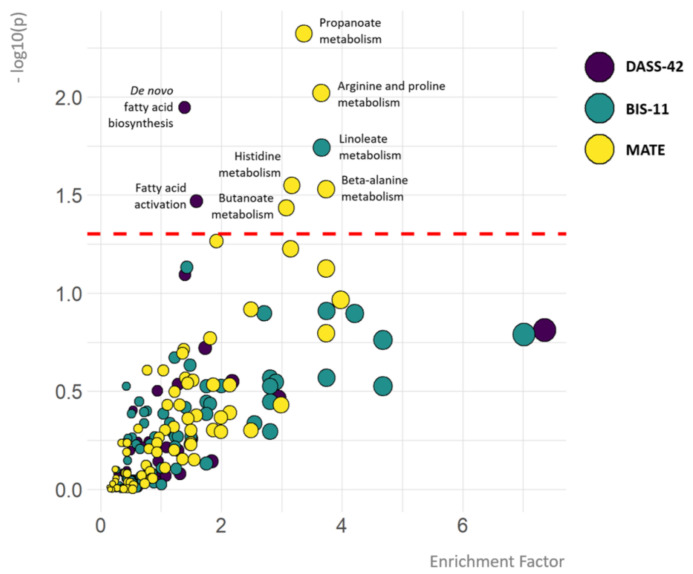
Metabolic pathways associated with depression, anxiety and stress, impulsivity and substance use. The colour of the circles represents the psychiatric outcome (DASS-42, BIS-11 or MATE-Q scores). The *y*-axis depicts the significance of the association between pathways and DASS-42, BIS-11 or MATE-Q scores. Cut-off *p* = 0.05 equals −log10(*p*-value) = 1.3. The *x*-axis and size of the circles depict the enrichment factor, which refers to the ratio between the number of significant pathway hits and the expected number of compound hits within the pathway (no cut-off value). Only the names of metabolic pathways significantly associated with psychiatric outcome measures are depicted.

The results of the mummichog pathway analysis of the metabolites significantly associated with DASS-42, BIS-11, and MATE-Q scores in the 200HIV cohort are shown in Figure 1.

**Figure 2:**
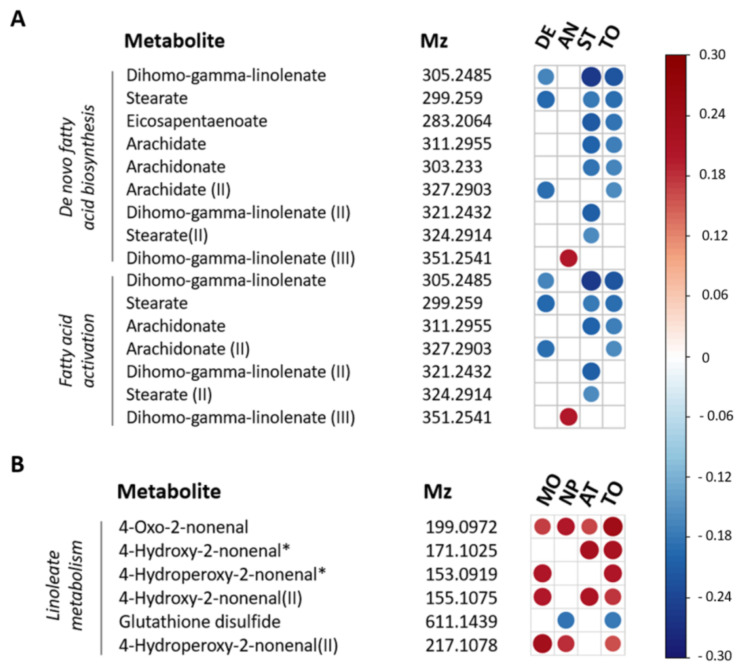
Spearman correlation plot for individual metabolites contributing to metabolic pathways associated with depression, anxiety and stress and impulsivity. (**A**) Metabolic features associated with DASS-42 scores involved in de novo fatty acid biosynthesis and fatty acid activation. (**B**) Metabolic features associated with BIS-11 scores and involved in linoleate metabolism. Strength and direction of the correlation coefficients are only shown for *p* < 0.05 (not FDR-corrected). The *m*/*z* values within each pathway are ordered based on *p*-values. *m*/*z* values referring to multiple significant pathways are depicted multiple times. II, III = multiple *m*/*z* values could be identified as the same metabolites. * = *m*/*z* value could be identified as multiple possible metabolites within a pathway, see Supplementary Figure S3 for all options. DE = depression, AN = anxiety, ST = stress, MO = motor impulsivity, NP = non planning impulsivity, AT = attentional impulsivity, TO = total DASS-42 (**A**) or BIS-11 score (**B**).

Pathways showing significant association with DASS total scores included de novo fatty acid biosynthesis (*p* = 0.01), and fatty acid activation (*p* = 0.03). The association between fatty acid related pathways and depression, anxiety and stress was mainly driven by the association between lower abundances of omega-6 (aradichonate and dihomo-γ-linolenate) and omega-3 (eicosapentaenoate) fatty acids and higher DASS-42 stress levels (Figure 2).

The only pathway showing significant association with impulsivity was linoleate metabolism (*p* = 0.02). Based on correlation coefficients, this was due to a positive association between levels of linoleate oxidation products and BIS-11 levels (Figure 2).

**Figure 3:**
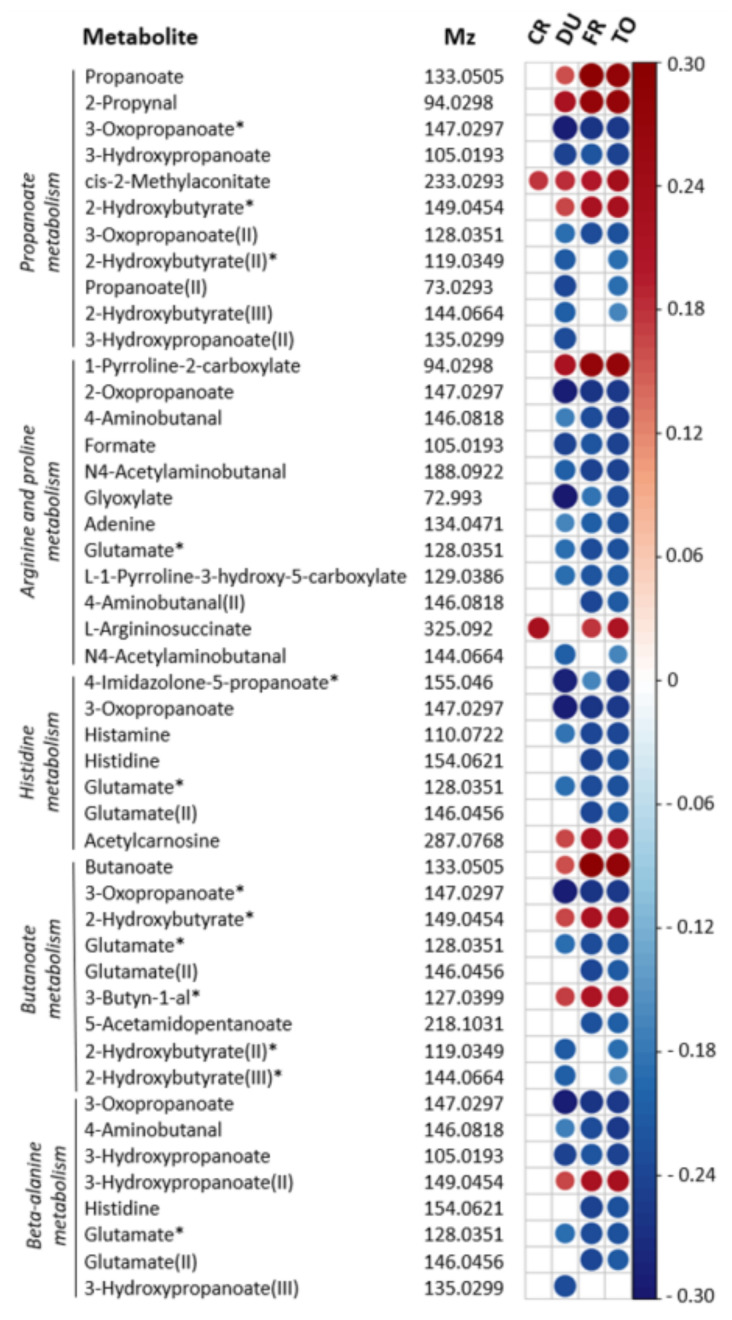
Spearman correlation plot for individual metabolites contributing to metabolic pathways associated with substance use. Metabolic features associated with MATE-Q scores and involved in propanoate metabolism, arginine and proline metabolism, histidine metabolism, butanoate metabolism and beta-alanine metabolism are depicted. Strength and direction of the correlation coefficients are only shown for *p* < 0.01 (not FDR-corrected). Results are corrected for age, gender and BMI. The *m*/*z* values referring to multiple significant pathways are depicted multiple times. I, II, III = multiple *m*/*z* values could be identified as the same metabolites. * = *m*/*z* value identified as multiple possible metabolites within a pathway, see Supplementary Figure S4 for all options. CR = craving (OCDS), DU = duration of substance use, FU = frequency of substance use, TO = total MATE score.

Pathways showing significant associations with MATE-Q scores were propanoate metabolism (*p* = 0.005), arginine and proline metabolism (*p* = 0.01), histidine metabolism (*p* = 0.03), butanoate metabolism (*p* = 0.04) and beta-alanine metabolism (*p* = 0.03). In the short chain fatty acid (SCFA) related pathways (e.g., propanoate and butanoate metabolism), propanoate and butanoate levels were both positively associated with MATE-Q scores. The association between arginine and proline metabolism and substance use was mainly driven by negative correlations between individual metabolites and MATE-Q scores, however, proline metabolite 1-pyrroline-2-carboxylate and arginine precursor L-argininosuccinate were both positively associated with MATE-Q scores. Importantly, across all pathways significantly associated with substance use, the effect was partly driven by a negative association between glutamate levels and MATE-Q scores (Figure 3).

## 4. Discussion

The current study explored associations between metabolic pathways and psychiatric symptomatology in PLWH. De novo fatty acid biosynthesis and fatty acid activation pathways were associated with depression, anxiety and stress, mainly driven by negative associations between fatty acids and DASS-42 scores. Linoleate metabolism was associated with impulsivity, an effect driven by a positive association between linoleate oxidation products and BIS-11 scores. Finally, butanoate, arginine and proline, histidine, butanoate and beta-alanine metabolism were associated with substance use in PLWH. Amongst the metabolites contributing to these associations were butanoate and propanoate, which were positively associated with MATE-Q scores, and glutamate, which was negatively associated with MATE-Q scores.

We identified three fatty acid-related pathways as potentially involved in HIV-related psychiatric symptomatology: de novo fatty acid biosynthesis and fatty acid activation were both associated with internalizing symptomatology, and linoleate metabolism was associated with externalizing symptomatology. Regarding internalizing symptomatology, lower abundances of dihomo-γ-linolenate, aradichonate and eicosapentaenoate were associated with higher DASS-42 levels. Regarding externalizing symptomatology, higher abundances of linoleate oxidation products were associated with higher BIS-11 scores. Linoleate, dihomo-gamma-linolenate, aradichonate and eicosapentaenoate are also known as polyunsaturated fatty acids (PUFAs). Looking in more detail at PUFA subclasses, it is important to note that dihomo-γ-linolenate and aradichonate are omega-6 PUFA’s that derive from linoleate, an essential omega-6 PUFA. In contrast with our findings, some studies have reported higher omega-6 PUFA levels in depression, generally explained by their presumed harmful pro-inflammatory effects [48,49,50,51,52]. However, in line with our results, a meta-analysis of 46 studies showed decreased levels of linoleate in patients suffering from major depressive disorder [53]. Previous literature on omega-6 levels and externalizing symptomatology is scarce and inconclusive: one study demonstrated reduced linoleate levels, but elevated dihomo-γ-linolenate levels in violent, impulsive male offenders with alcohol use disorder [54].

Based on our untargeted metabolomics study, no conclusion can be drawn regarding the total omega-3 versus omega-6 PUFA ratio and the occurrence of depression or impulsivity. However, our results underline the importance of PUFAs in a broad spectre of internalizing as well as externalizing symptomatology. The hypothesis-free nature of this study confirms previous reports on this association from studies using targeted analyses. In addition, we report this association for the first time in PLWH. Interestingly, previous research in PLWH has demonstrated reduced PUFA levels in HIV-infected individuals, and a negative association between PUFA levels and markers of monocyte activation [55].

In depression and impulsivity research, a pathophysiological role of omega-3 PUFA depletion has been suggested [56,57]. PUFA levels mainly depend on the dietary intake of fish and vegetable oils. Interestingly, PUFAs are known to pass the blood brain barrier and regulate numerous functions within the brain. Arachidonate for example, is known to modulate the endocannabinoid system and influence brain inflammation [58]. Several hypotheses could explain the association between depression, impulsivity and PUFA-levels. It could be speculated that the variation in dietary PUFA-intake is a causal factor contributing to symptoms of depression and impulsivity [54]. Alternatively, symptoms of depression or impulsivity might influence patient’s food intake, and thereby cause PUFA level alterations. Future experimental studies should explore causality of the observed associations between PUFA levels and psychiatric symptomatology in PLWH.

SCFA-related pathways (specifically propanoate and butanoate metabolism) were associated with substance use in our PLWH cohort. Higher propanoate and butanoate levels were associated with higher MATE-Q scores. This association between SCFA-pathways and substance use has not been reported in previous studies. SCFA’s propanoate and butanoate are produced by the intestinal microbiome. Although most of the SCFAs are used as energy source by the intestine, a part is transported to the liver and reaches the systemic circulation. SCFAs are known to modulate immune function, and influence brain physiology by crossing the blood-brain barrier (BBB), stimulating the vagal nerve and modulating BBB integrity [59]. Furthermore, SCFAs can inhibit histone deacetylase (HDAC), subsequently stimulating gene expression [60].

Several mechanisms could explain the observed relationship between substance use and SCFAs. For instance, substance use is known to cause reduced intestinal microbial diversity (dysbiosis), which can result in SCFA alterations [61]. Vice versa, SCFA alterations induced by dysbiosis are known to affect brain function [59], and are therefore speculated to affect cognition and emotion [62], which might also contribute to the occurrence of substance use [61]. Animal data support this hypothesis, by demonstrating that antibiotic treatment-induced gut dysbiosis results in enhanced sensitivity to rewarding effects of cocaine in mice [63]. Supplementation with SCFAs reversed these effects [63]. Finally, the relationship between SCFAs and substance use might also be explained by shared associations with other factors, like sexual behaviour and diet [64].

It is unknown if the observed relationship between substance use and SCFAs is HIV-specific. HIV infection and the use of cART are known to influence the microbiome. In PLWH, gut dysbiosis and higher levels of SCFAs have been demonstrated [65]. Furthermore, several studies have shown that the immunological effects of SCFAs depend on the immunological milieu [66]. Therefore, future research should further assess if HIV-infection modulates the relation between substance use and SCFAs.

A negative association between glutamate levels and MATE-Q scores contributed to several pathways significantly associated with substance use. Glutamate, a non-essential amino acid and N-methyl-D-aspartate receptor agonist, is the major excitatory neurotransmitter in the brain [67]. The BBB prevents transport of glutamate from the peripheral circulation into the brain [67]. However, BBB transporters actively transport glutamate from the brain back into the circulation [67]. Glutamate concentrations in the peripheral circulation partly depend on this active removal of glutamate from the brain. It has been demonstrated that peripheral glutamate levels significantly correlate with brain glutamate levels, with a medium effect size [68]. Glutamate signaling is known to be involved in various phases of the addiction cycle [69]. In addition, HIV-1 envelope glycoprotein gp120 is known to directly affect glutamate uptake and release [70,71]. Alterations in glutamate signaling may therefore be of particular interest in relation to substance use in PLWH. Future studies should further explore potential causal mechanisms involved, as well as therapeutic potential of glutamate modulators, like acamprosate that is frequently prescribed in alcohol use disorders [72].

This study should be considered in the light of several strengths and limitations. First, we applied an innovative data-driven, hypothesis free approach, in a substantially large sample, which enables the exploration of previously unknown pathophysiological mechanisms. Second, we examined both internalizing and externalizing psychiatric symptoms simultaneously in order to take into account the frequent overlap between psychiatric symptomatology.

It is important to note that the field of metabolomics is evolving rapidly. As a result, the risk of metabolite misidentification cannot be fully eliminated at the current stage [73]. Moreover, current metabolomic technologies capture only a small portion of the metabolome, which results in potential bias. Perhaps yet unidentified metabolites play an important role in the pathophysiology of HIV-related psychiatric symptomatology. Also, peripheral metabolite abundances do not necessarily reflect concentrations in the brain. Therefore, future metabolomics studies using cerebrospinal fluid, brain tissue, as well as peripheral blood are needed. Similarly, linking metabolome findings with neural function would be an essential next step to further elucidate causal mechanisms linking metabolome findings with psychiatric symptomatology. Furthermore, the cross-sectional design of this study limits causal inferences about the observed associations. Multi-omics and experimental (e.g., animal or organoid) studies are needed to elucidate if a causal relationship between BCAA metabolism and psychiatric symptoms exists.

Another limitation is related to the phenotype measures applied here. Though generally considered reliable, the use of self-report questionnaires to assess psychiatric symptom levels has some limitations [74]. For instance, it may result in social desirability bias (due to the tendency to respond in a socially acceptable way) or response bias (due to the tendency to respond in a certain way regardless of the question) [74]. However, the self-report measures applied here have the advantage of providing continuous measures of psychiatric symptomatology, covering the full spectrum of symptom severity, and thus increasing ecological validity and statistical power. Future studies should also use diagnostic interviews and behavioral tasks, for instance based on the research domain criteria (RDoC) domains to further study transdiagnostic metabolomic mechanisms in psychiatric symptoms in PLWH and other clinical populations [75].

In conclusion, in this transdiagnostic, hypothesis-free metabolomics study, we observed an association between fatty acid-related pathways and both depressive as well as impulsive symptomatology in PLWH. This extends previous findings on the role of fatty acids in psychiatric disorders, and further points at the intimate link between physical and mental disorders, specifically towards potential metabolic and immunological mechanisms contributing to psychiatric symptomatology. Furthermore, we identified SCFA- and glutamate-related pathways as potential mechanisms involved in substance use in PLWH. The innovative metabolome approach applied here, might pave the way towards linking omics data with psychopathology. Future studies should explore potential causal mechanisms involved and investigate whether these findings generalize to other populations without HIV.

## Figures and Tables

**Table 1:** Patient characteristics (*n* = 157).

Demographic Characteristics		Psychiatric Characteristics	
**Age, mean (SD), y**	51.5 (10.7)	**DASS-42**	
**Females, No. (%)**	14 (8.9)	Total score, median (IQR)	13 (24.6)
**BMI, mean (SD), kg/m^2^**	24.6 (3.8)	Total score > 60, No. (%)	6 (3.8)
**Civil status**		Depression, median (IQR)	4 (10.0)
Living alone, No. (%)	56 (25.7)	Anxiety, median (IQR)	2 (5.0)
Living with partner/family, No. (%)	95 (60.5)	Stress, median (IQR)	7 (9.0)
Other, No. (%)	6 (3.8)	**BIS-11**	
**Educational level achieved**		Total score, mean (SD)	60.0 (8.8)
No certificate, No. (%)	18 (11.5)	Motor impulsivity, mean (SD)	20.4 (3.4)
Secondary school, No. (%)	25 (15.9)	Nonplanning impulsivity, mean (SD)	24.0 (4.9)
Vocational training, No. (%)	60 (38.2)	Attentional impulsivity, mean (SD)	15.6 (3.1)
Higher education, No. (%)	54 (34.4)	**MATE-Q**	
**HIV-related characteristics**		Alcohol use, No. (%)	109 (69.4)
**Time since HIV diagnosis, mean (SD), y**	9.9 (6.6)	Smoking, No. (%)	46 (29.3)
**Way of transmission**		Cannabis use, No. (%)	31 (19.7)
MSM, No. (%)	114 (72.6)	XTC use, No. (%)	25 (15.9)
Heterosexual contact, No. (%)	7 (4.5)	Cocaïne use, No. (%)	5 (3.2)
Intravenous drug use, No. (%)	3 (1.9)	OCDS-5, median (IQR)	0 (4.0)
Other, No. (%)	33 (21.0)	**Use of psychiatric medication**	
**Nadir CD4+ count, mean (SD), cells/μL**	272.5 (170.6)	Benzodiazepines, No. (%)	12 (7.6)
**Current CD4+ count, mean (SD), cells/μL**	692.6 (258.6)	SSRI’s, No. (%)	8 (5.1)
**Time on cART, mean (SD), y**	8.6 (6.2)	TCA’s, No. (%)	2 (1.3)
**ARV classes**		Antipsychotics, No. (%)	6 (3.8)
NRTI, No. (%)	149 (94.9)		
INSTI, No. (%)	102 (65.0)		
NtRTI, No. (%)	70 (44.6)		
NNRTI, No. (%)	48 (30.6)		
PI, No. (%)	25 (15.9)		

ARV = antiretroviral drug, BIS-11 = Barratt Impulsiveness Scale, BMI = body mass index, cART = combination antiretroviral therapy, INSTI = integrase inhibitor, IDU = intravenous drug use, MSM = men who have sex with men, NRTI = nucleoside reverse transcriptase inhibitor, NNRTI = non-nucleoside reverse transcriptase inhibitor, NtRTI = nucleotide reverse transcriptase inhibitor, PI = protease inhibitor.

## Data Availability

The data presented in this study are available on request from the corresponding author. The data are not publicly available due to privacy reasons.

## Supplementary Materials

The following are available online at https://www.mdpi.com/article/10.3390/jcm10235466/s1, Figure S1: Study flow-chart; Table S1: Metabolomic pathways significantly associated with BIS-11, DASS-42 and MATE-Q scores; Table S2: Annotation of metabolic features contributing to the association between DASS-42 scores and the pathways of de novo fatty acid biosynthesis and fatty acid activation; Figure S2: Metabolic network associated with depression, anxiety and stress in the 200HIV cohort; Table S3: Annotation of metabolic features contributing to the association between BIS-11 scores and the pathway of linoleic acid metabolism; Figure S3: Metabolic network associated with impulsivity in the 200HIV cohort; Table S4: Annotation of metabolic features contributing to the association between MATE-Q scores and the pathways of propanoate metabolism, arginine and proline metabolism, histidine metabolism, butanoate metabolism and β-alanine metabolism; Figure S4: Metabolic network associated with substance use in the 200HIV cohort.
