# Supplementary material for: Unbiased Metabolomics Links Fatty Acid Pathways to Psychiatric Symptoms in People Living with HIV
Source: J Clin Med. 2021 Nov 23;10(23):5466. doi: 10.3390/jcm10235466 (PMC8658345; doi:10.3390/jcm10235466)
Supplement: Supplementary file 1 [file jcm-10-05466-s001.zip › jcm-1448910-supplementary.pdf]

Supplementary Materials

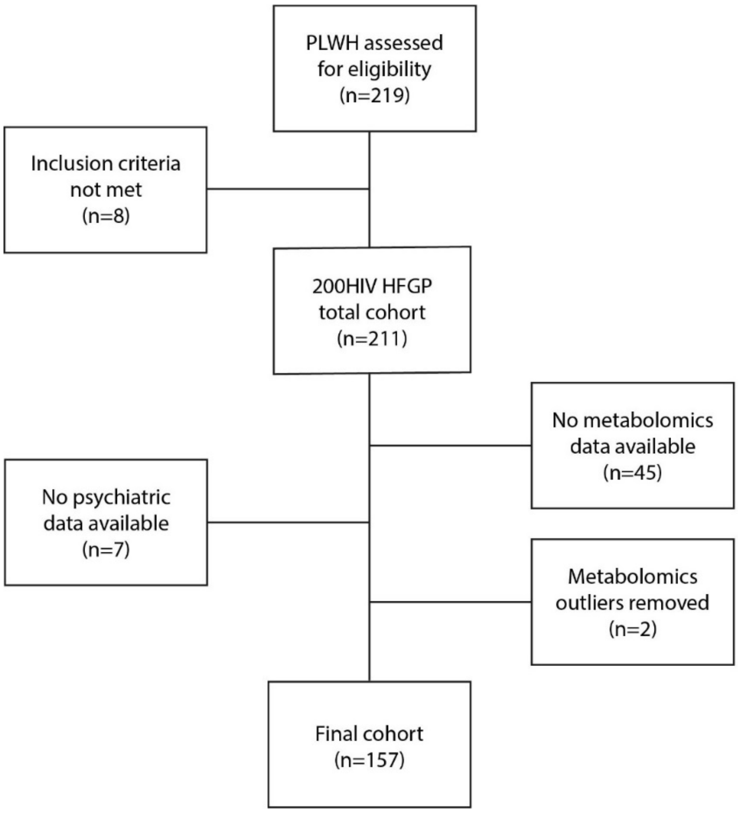

Supplementary Figure S1. Study flow-chart

Supplementary Table S1. Metabolomic pathways significantly associated with BIS-11, DASS-42 and MATE-Q scores, ordered based on significance.

Metabolomic pathway	Variable	Enrichment	$-\log_{10}$ p-value
Propanoate metabolism	MATE-Q	3.371382	2.322302
Arginine and proline metabolism	MATE-Q	3.649635	2.02192
Histidine metabolism	MATE-Q	3.166991	1.54892
Beta-alanine metabolism	MATE-Q	3.732457	1.530959
Butanoate metabolism	MATE-Q	3.07395	1.433786
De novo fatty acid biosynthesis	DASS-42	1.387809	1.945425
Fatty acid oxidation	DASS-42	1.590331	1.467883
Linoleate metabolism	BIS-11	1.387809	1.945425

Supplementary Table S2. Annotation of metabolic features contributing to the association between DASS-42 scores and the pathways of de novo fatty acid biosynthesis and fatty acid activation.

Mz	KEGG	Metabolite name
***De novo* fatty acid biosynthesis**		
305.2485	C03242	Dihomo-gamma-linolenate
299.259	C01530	Stearate
283.2064	C06428	Eicosapentaenoate
311.2955	C06425	Arachidate
303.233	C00219	Arachidonate
327.2903	C06425	Arachidate
321.2432	C03242	Dihomo-gamma-linolenate (II)
324.2914	C01530	Stearate (II)
351.2541	C03242	Dihomo-gamma-linolenate (III)
**Fatty acid activation**		
305.2485	C03242	Dihomo-gamma-linolenate
299.259	C01530	Stearate
311.2955	C06425	Arachidate
327.2903	C06425	Arachidate (II)
321.2432	C03242	Dihomo-gamma-linolenate (II)
324.2914	C01530	Stearate (II)
351.2541	C03242	Dihomo-gamma-linolenate (III)

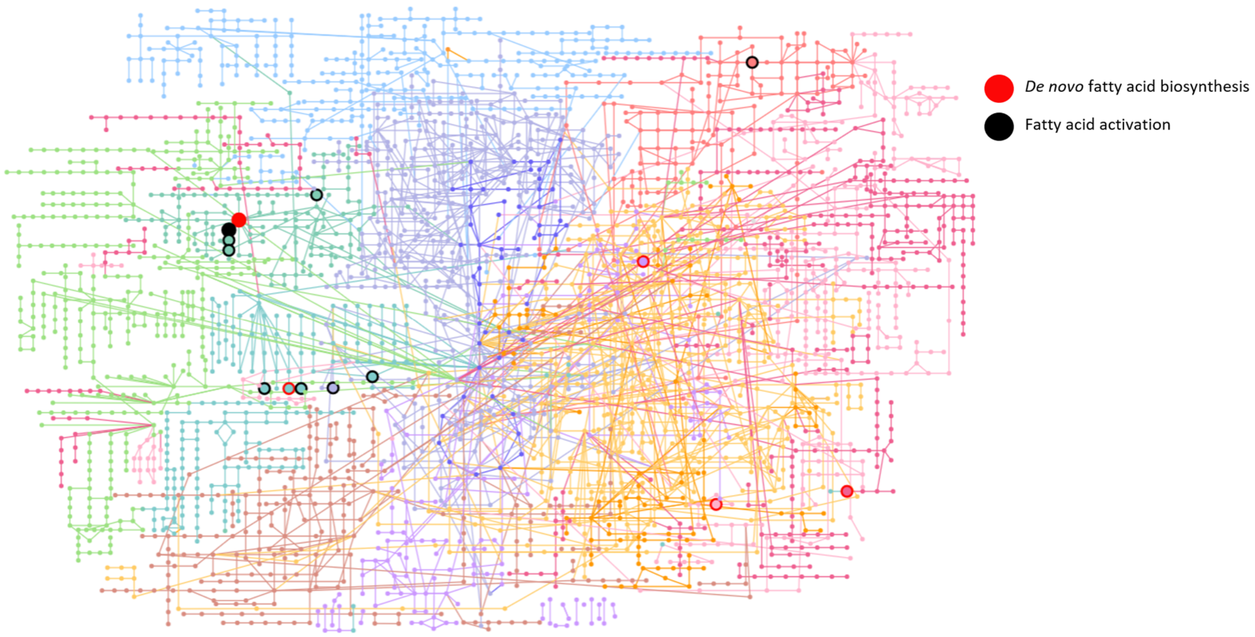

Supplementary Figure S2. Metabolic network associated with depression, anxiety and stress in the 200HIV cohort. For the metabolic pathways associated with DASS-42 scores, the metabolites that were detected in the 200HIV cohort are highlighted. Solid circles indicate metabolites significantly associated with DASS-42 scores, whereas empty circles represent metabolites that were detected but not significantly associated with DASS-42 scores. The colour of the circles represents the corresponding pathway.

Supplementary Table S3. Annotation of metabolic features contributing to the association between BIS-11 scores and the pathway of linoleic acid metabolism.

Mz	KEGG	Metabolite name
Linoleic acid metabolism		
199.0972	CE2577	4-Oxo-2-nonenal
171.1025	CE2006/CE2576/CE6506	4-Hydroxy-2-nonenal/4-Hydroperoxy-2-nonenal/3,4-Epoxynonanal
153.0919	CE2576/CE2577	4-Hydroperoxy-2-nonenal(II)/4-Oxo-2-nonenal(II)
155.1075	CE2006/C04717/C14827/CE6506	4-Hydroxy-2-nonenal(III)/13S-Hydroperoxy-9Z,11E-octadecadienoate/(9S,10E,12Z)-9-Hydroperoxy-10,12-octadecadienoate/3,4-Epoxynonanal
611.1439	C00127	Glutathione disulfide
217.1078	CE2576	4-Hydroperoxy-2-nonenal

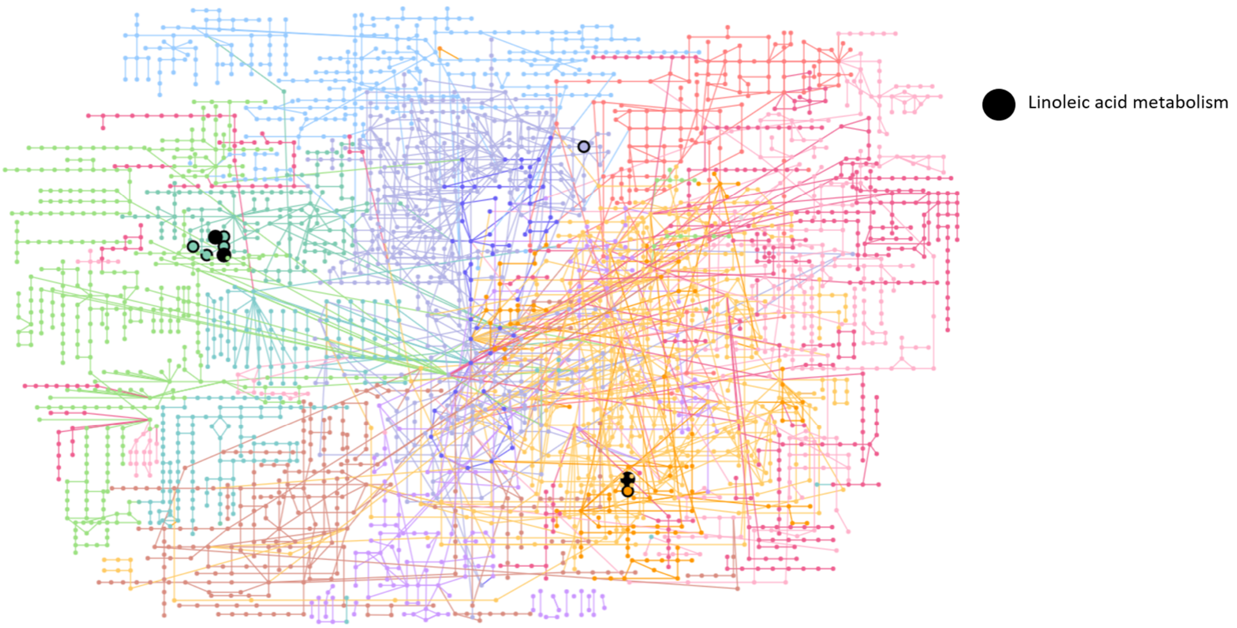

Supplementary Figure S3. Metabolic network associated with impulsivity in the 200HIV cohort. For the metabolic pathway associated with BIS-11 scores, the metabolites that were detected in the 200HIV cohort are highlighted. Solid circles indicate metabolites significantly associated with BIS-11 scores, whereas empty circles represent metabolites that were detected but not significantly associated with BIS-11 scores.

Supplementary Table S4. Annotation of metabolic features contributing to the association between MATE-Q scores and the pathways of propanoate metabolism, arginine and proline metabolism, histidine metabolism, butanoate metabolism and beta-alanine metabolism.

Mz	KEGG	Metabolite name
**Propanoate metabolism**		
133.0505	C00163	Propanoate
94.0298	C05985	2-Propynal
147.0297	C00222/C06002/C00109/ C00164	3-Oxopropanoate/(S)-Methylmalonate semialdehyde/ 2-Oxobutanoate/3-Oxobutanoate
105.0193	C01013	3-Hydroxypropanoate
233.0293	C04225	cis-2-Methylaconitate
149.0454	C05984/C01013	2-Hydroxybutyrate/3-Hydroxypropanoate(II)
128.0351	C00222	3-Oxopropanoate(II)
119.0349	C05984/C00163	2-Hydroxybutyrate(II)/Propanoate(II)
73.0293	C00163	Propanoate(III)
144.0664	C05984	2-Hydroxybutyrate(III)
135.0299	C01013	3-Hydroxypropanoate(III)
**Arginine and proline metabolism**		
94.0298	C03564	1-Pyrroline-2-carboxylate
147.0297	C00022	2-Oxopropanoate
146.0818	C00555	4-Aminobutanal
105.0193	C00058	Formate
188.0922	C05936	N4-Acetylaminobutanal
72.993	C00048	Glyoxylate
134.0471	C00147	Adenine
128.0351	C00025/C00022/C03912/ C03564/C04281/C05938	Glutamate/2-Oxopropanoate/1-Pyrroline-5-carboxylate/1-Pyrroline-2-carboxylate/3-Hydroxy-L-1-pyrroline-5-carboxylate/L-4-Hydroxyglutamate semialdehyde
129.0386	C04281	L-1-Pyrroline-3-hydroxy-5-carboxylate
146.0818	C00555	4-Aminobutanal
325.092	C03406	L-Argininosuccinate
144.0664	C05936	N4-Acetylaminobutanal
**Histidine metabolism**		
155.046	C03680/C00439/C05828/ C05130	4-Imidazolone-5-propanoate/N-Formimino-L-glutamate/1-Methylimidazole-4-acetate/Imidazole-4-acetaldehyde
147.0297	C00222	3-Oxopropanoate
110.0722	C00388	Histamine
154.0621	C00135	Histidine
128.0351	C00025/C00222	Glutamate/3-Oxopropanoate(II)
146.0456	C00025	Glutamate(II)
287.0768	CE2065	Acetylcarnosine
**Beta-alanine metabolism**		
147.0297	C00222	3-Oxopropanoate
146.0818	C00555	4-Aminobutanal
105.0193	C01013	3-Hydroxypropanoate
149.0454	C01013	3-Hydroxypropanoate(II)
154.0621	C00135	Histidine
128.0351	C00025/C00222	Glutamate/3-Oxopropanoate(II)
146.0456	C00025	Glutamate(II)
135.0299	C01013	3-Hydroxypropanoate(III)
**Butanoate metabolism**		
133.0505	C00246	Butanoate

147.0297	C00022/C00232/C00109/C06010/C00164	3-Oxopropanoate/4-Oxobutanoate/2-Oxobutanoate/(S)-2-Acetolactate/3-Oxobutanoate
149.0454	C05984/C01089	2-Hydroxybutyrate/(R)-3-Hydroxybutanoate
128.0351	C00025/C00022	Glutamate/3-Oxopropanoate
146.0456	C00025	Glutamate
127.0399	C06145/C06006	3-Butyn-1-al/(S)-2-Aceto-2-hydroxybutanoate
218.1031	C03087	5-Acetamidopentanoate
119.0349	C05984/C01089	2-Hydroxybutyrate/(R)-3-Hydroxybutanoate
144.0664	C05984/C01089	2-Hydroxybutyrate/(R)-3-Hydroxybutanoate

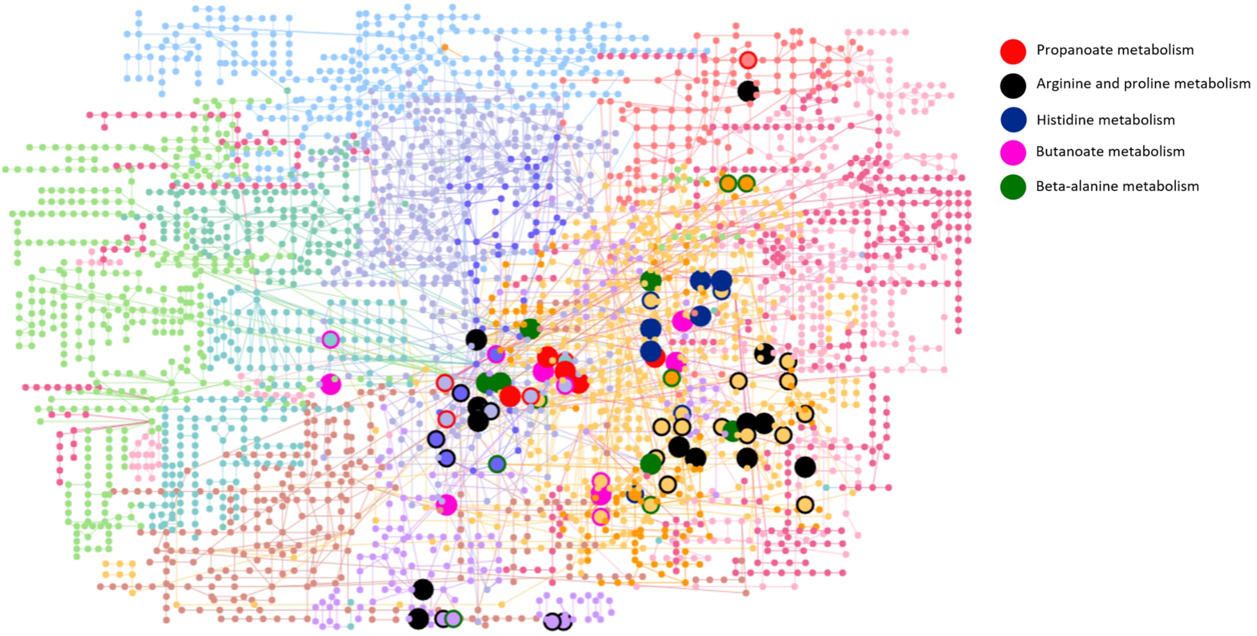

Supplementary Figure S4. Metabolic network associated with substance use in the 200HIV cohort. For all metabolic pathways associated with MATE-Q scores, the metabolites that were detected were detected in the 200HIV cohort are highlighted. Solid circles indicate metabolites significantly associated with MATE-Q scores, whereas empty circles represent metabolites that were detected but not significantly associated with MATE-Q scores. The colour of the circles represents the corresponding pathway.
